# An investigation of ribosomal protein L10 gene in autism spectrum disorders

**DOI:** 10.1186/1471-2350-10-7

**Published:** 2009-01-23

**Authors:** Xiaohong Gong, Richard Delorme, Fabien Fauchereau, Christelle M Durand, Pauline Chaste, Catalina Betancur, Hany Goubran-Botros, Gudrun Nygren, Henrik Anckarsäter, Maria Rastam, I Carina Gillberg, Svenny Kopp, Marie-Christine Mouren-Simeoni, Christopher Gillberg, Marion Leboyer, Thomas Bourgeron

**Affiliations:** 1 Human Genetics and Cognitive Functions, CNRS URA 2182 "Genes, Synapses and Cognition", Institut Pasteur, Paris, France; 2 State Key Laboratory of Genetic Engineering, School of Life Science, Fudan University, Shanghai, PR China; 3 Université Denis Diderot Paris 7, Paris, France; 4 INSERM U513, Créteil, France; 5 Université Paris XII, Faculté de Médecine, Créteil, France; 6 Department of Child and Adolescent Psychiatry, Göteborg University, Göteborg, Sweden; 7 AP-HP, Hôpital Robert Debré, Service de Psychopathologie de l'Enfant et de l'Adolescent, Paris, France; 8 Institute of Child Health, London, UK; 9 AP-HP, Groupe Hospitalier Henri Mondor – Albert Chenevier, Department of Psychiatry, Créteil, France

## Abstract

**Background:**

Autism spectrum disorders (ASD) are severe neurodevelopmental disorders with the male:female ratio of 4:1, implying the contribution of X chromosome genetic factors to the susceptibility of ASD. The ribosomal protein L10 (RPL10) gene, located on chromosome Xq28, codes for a key protein in assembling large ribosomal subunit and protein synthesis. Two non-synonymous mutations of *RPL10*, L206M and H213Q, were identified in four boys with ASD. Moreover, functional studies of mutant RPL10 in yeast exhibited aberrant ribosomal profiles. These results provided a novel aspect of disease mechanisms for autism – aberrant processes of ribosome biosynthesis and translation. To confirm these initial findings, we re-sequenced *RPL10 *exons and quantified mRNA transcript level of *RPL10 *in our samples.

**Methods:**

141 individuals with ASD were recruited in this study. All *RPL10 *exons and flanking junctions were sequenced. Furthermore, mRNA transcript level of *RPL10 *was quantified in B lymphoblastoid cell lines (BLCL) of 48 patients and 27 controls using the method of SYBR Green quantitative PCR. Two sets of primer pairs were used to quantify the mRNA expression level of *RPL10*: RPL10-A and RPL10-B.

**Results:**

No non-synonymous mutations were detected in our cohort. Male controls showed similar transcript level of RPL10 compared with female controls (RPL10-A, U = 81, P = 0.7; RPL10-B, U = 61.5, P = 0.2). We did not observe any significant difference in RPL10 transcript levels between cases and controls (RPL10-A, U = 531, P = 0.2; RPL10-B, U = 607.5, P = 0.7).

**Conclusion:**

Our results suggest that RPL10 has no major effect on the susceptibility to ASD.

## Background

Autism spectrum disorders (ASD) are complex neurobehavioral disorders characterized by impaired social interaction and language development and by repetitive and stereotyped behaviors and interests. Twin and family studies indicate that genetic factors contribute to the susceptibility to ASD[1,2]. However, only 10–25% of cases of autism harbour identified chromosome abnormalities and/or present with genetic syndromes; the remaining 90% are idiopathic[3]. The male:female ratio of autism is approximately 4:1, implying the contribution of X chromosome genetic factors to the susceptibility of ASD.

Ribosomal protein L10 (RPL10), also called QM, is a highly conserved component of the large ribosome subunit (60s) that plays a crucial role in protein synthesis[4]. In humans, the *RPL10 *gene is located on chromosome Xq28, within a candidate region for ASD[5]. Recently, Klauck *et al. *identified two non-synonymous mutations, L206M and H213Q, in the C-terminal domain of *RPL10 *in four boys with ASD from two independent families. Furthermore, functional studies using yeast strains expressing human mutant RPL10 cDNAs exhibited aberrant ribosomal profiles, suggesting that the mutations may actually have an effect on translation[6]. High expression of RPL10 was observed in mouse hippocampus, an important brain site for learning and memory, which are impaired in autism[7]. To investigate whether *RPL10 *is involved in the pathogenesis of autism, we sequenced all *RPL10 *exons and quantified RPL10 mRNA level in patients with ASD and controls.

## Methods

### Subjects

A total of 141 individuals with ASD were recruited by the Paris Autism Research International Sibpair (PARIS) study. All probands met DSM-IV diagnostic criteria for autism, Asperger syndrome or pervasive developmental disorder not otherwise specified (PDD-NOS)[8]. The patients were evaluated by experienced psychiatrists or child neurologists and assessed with the Autism Diagnostic Interview-Revised (ADI-R) [9] or the Asperger Syndrome Diagnostic Interview (ASDI) [10]. Totally, 129 subjects met full criteria for autistic disorder, 6 for Asperger Syndrome and 6 for PDD-NOS. 109 patients showed low IQ (<70). Laboratory tests to rule out medical causes of autism included standard karyotyping, fragile X testing, and metabolic screening. Brain imaging and EEG were performed when possible. Patients diagnosed with medical disorders such as fragile X syndrome or chromosomal abnormalities were excluded from the study. Of 141 patients, 88 were selected from the families with X chromosome inactivation skewing (XCI) from 289 ASD families based on the previous study[8]. The other 53 patients were from multiplex families with random XCI. There were 101 males and 40 females (78 subjects from multiplex families and 63 sporadic cases). All individuals were of European descent, except 5 sub-Saharan Africans, and 3 of mixed ethnicity. The study was approved by the research ethics boards of the collaborating institutions. Informed consent was obtained from all families participating in the study.

### Sequencing

All *RPL10 *exons and flanking junctions were amplified using the primers described previously[6]. Amplicons were directly sequenced using the Big Dye version 3.1 in ABI 3100 sequencers (Applied Biosystems, Foster City, CA)

### Real-Time Quantitative PCR

A sample of 48 patients (34 males and 14 females, of which 35 patients IQ<70) and 27 controls (15 males and 12 females, all IQ>90) were available for B lymphoblastoid cell lines (BLCL). Total RNA was isolated from BLCL using the NucleoSpin^® ^RNA II kit. Oligo(dT)-primed cDNA prepared from 5 μg of BLCL RNA using Superscript II (Invitrogen, Carlsbad, CA) was used as template for quantitative PCR with SYBR Green on an ABI PRISM 7500 instrument (Applied Biosystems, Foster City, CA). Two sets of primer pairs were used to quantify the mRNA expression level of *RPL10*: RPL10-A, spanning exons 4 and 5, and RPL10-B, spanning exons 6 and 7. The forward primer of RPL10-A was 5'-ATA TGA GCA GCT GTC CTC TGA AG-3' and the reverse was 5'-CCA TCT TTG CCA CAA CTT TTT ACC-3'. The forward primer of RPL10-B was 5'-AGA ACA AGG AGC ATG TGA TTG AG-3' and the reverse was 5'-CTT CTT TGA GAT GTG GAT CTT CTG-3'. *GAPDH *was used as an endogenous control. The forward primer of *GAPDH *was 5'-GAT GAC ATC AAG AAG GTG GTG-3' and the reverse was 5'-GTC ATA CCA GGA AAT GAG CTT G-3'. The efficiencies of three primer sets were measured using a dilution series of cDNA. The raw threshold cycle (Ct) values were converted to linear form REL (relative expression level) by the 2^-ΔΔCt ^method to quantify the relative gene expression[11]. The REL of each transcript was normalized to *GAPDH *and relative to the mean Ct value of control males. All the reactions were performed in triplicate. Melting curves were analyzed for each reaction to ensure the specificity of the amplicons.

### Statistics

Statistic significance was calculated for *RPL10 *mRNA transcript levels of different groups using non parametric Mann-Whitney U-test in SPSS10.0. The significance lever for all statistical tests was P < 0.05.

## Results

**Figure 1:**
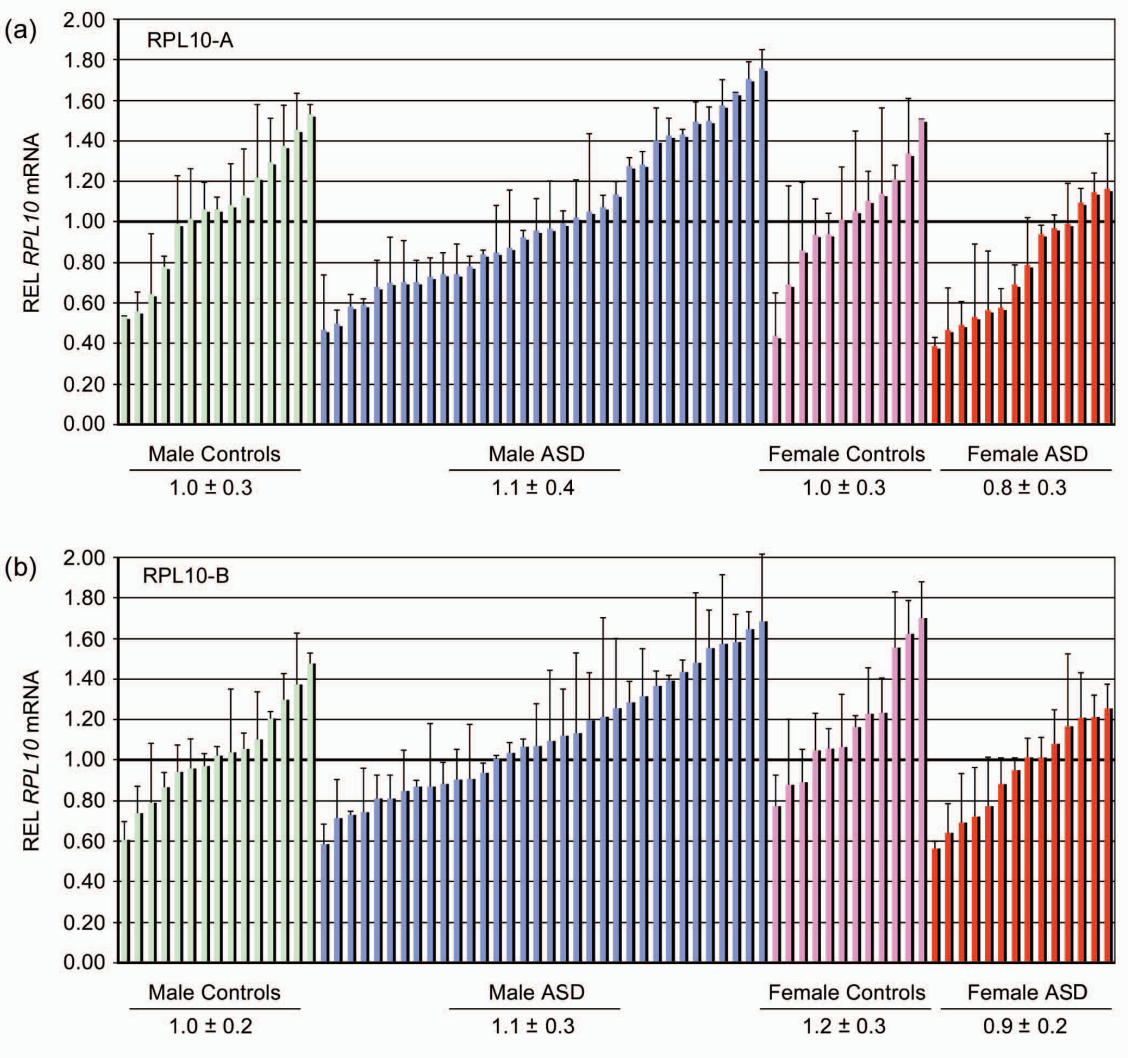
**Quantitative RT-PCR of RPL10 in patients with ASD and controls**. The level of RPL10 mRNA was quantified with two sets of primer pairs RPL10-A (a) and RPL10-B (b). Each column represents the value of relative expression level (REL) of an individual. The horizontal bar at 1 indicates the mean REL of male controls used as a calibrator. Four subgroups are shown in different colours. The REL value of each subgroup is expressed as mean ± SD.

No non-synonymous mutations were detected in our cohort. The mRNA level of *RPL10 *was compared in four subgroups: male controls, male ASD, female controls and female ASD using two sets of primer pairs RPL10-A and RPL10-B (Figure 1). In controls, the transcript level of RPL10 was not significantly different between males and females (RPL10-A, U = 81, P = 0.7; RPL10-B, U = 61.5, P = 0.2). When cases and controls were compared, we did not observe any significant difference in *RPL10 *transcript levels (RPL10-A, U = 531, P = 0.2; RPL10-B, U = 607.5, P = 0.7). Female cases had a somewhat lower expression level of *RPL10 *compared with female controls (RPL10-A, U = 49, P = 0.06; RPL10-B, U = 47.5, P = 0.07). However, this trend disappeared after correction for multiple tests.

## Discussion

*RPL10 *belongs to the L10e family of ribosomal proteins and is hypothesized to be necessary for ribosome assembling and function. The identification of two missense mutations in highly conserved positions of *RPL10 *across species and functional studies in yeast strains expressing human wild-type/mutant RPL10 cDNAs suggested a novel aspect of disease mechanisms for autism – aberrant processes of ribosome biosynthesis and translation. To confirm these initial findings, we sequenced all RPL10 exons and flanking junctions in 141 ASD patients. No missense mutation was identified. We performed this study in European population as well as Klauck *et al*[6] in order to exclude the possible sample stratification. Other factors, such as the sample size and highly genetic heterogeneity of ASD, should be considered when explaining the absence of *RPL10 *mutations in our sample. To further address the expression issue, mRNA transcript level of RPL10 was quantified in 48 patients and 27 controls using the method of SYBR Green quantitative PCR. Male controls showed the same transcript level of *RPL10 *compared with female controls, supporting the evidence that *RPL10 *is subject to X chromosome inactivation [12]. No statistical significance in expression level was found between cases and controls, suggesting there was no difference in RPL10 expression between two groups. Taken together, our study could not confirm the association between *RPL10 *and ASD.

Three limitations should be considered in this study. First, our sample of patients screened for *RPL10 *(n = 141, 101 males and 40 females) was smaller compared to the previous study (n = 345, 268 males and 77 females)[6]. We had 56% of chance to detect at least one mutation in our sample. Our previous study indicated XCI profile could be a useful criteria to prioritize families for mutation screening of X-linked candidate genes in ASD, so we selected 88 individuals from the families with XCI skewing (≥ 70:30) and 53 patients from multiplex families for mutation screening of RPL10[8]. However, we did not detect any functional mutations. Second, our mutation screening was restricted to exons and therefore was not appropriate to detect the presence of variants altering the expression of RPL10 in promoter regions or other regulatory regions. However, we recently performed a high-throughput genotyping of 91 patients from this sample using the Illumina human1M-duo beadchip, and we could not detect any genomic imbalance within or close to *RPL10 (unpublished data)*. Thirdly, we performed the quantification of RPL10 mRNA level in B lymphoblastoid cell lines and therefore we could have missed alterations specific to brain. Thus, further studies on other bigger samples are warranted and the promoter regions and other regulatory regions should be investigated.

## Conclusion

The present study did not find any non-synonymous mutations in our cohort, neither abnormal *RPL10 *transcript levels in ASD patients compared to controls, suggesting that *RPL10 *has no major effect on the susceptibility to ASD.

## Competing interests

The authors declare that they have no competing interests.

## Authors' contributions

XG carried out sequencing and quantitative PCR testing, and contributed to manuscript writing. RD performed the statistical analysis. FF aided in quantitative PCR testing. CMD, PC, and HGB aided in sample storage and preparations. CB, GN, HA, MR, ICG, SK, MCMS, CG and ML contributed to the sample collection. TB participated in the design of the study and manuscript writing.

## Pre-publication history

The pre-publication history for this paper can be accessed here:


